# Natural therapeutics for urinary tract infections—a review

**DOI:** 10.1186/s43094-020-00086-2

**Published:** 2020-09-18

**Authors:** Sarita Das

**Affiliations:** Department of Botany, Berhampur University, Bhanja Bihar, Berhampur, Orissa 760007 India

**Keywords:** Cranberry, Proanthocyanins, PAC, Urinary tract infections, Uropathogenic *Escherichia coli*, UTI, UPEC

## Abstract

**Background:**

The recurrence of the urinary tract infections (UTI), following the antibiotic treatments suggests the pathogen’s resistance to conventional antibiotics. This calls for the exploration of an alternative therapy.

**Main body:**

The anti-uropathogenic and bactericidal activity of many plant extracts was reported by many researchers, which involves only preliminary antibacterial studies using different basic techniques like disk diffusion, agar well diffusion, or minimum inhibitory concentration (MIC) of the crude plant extracts, but reports on the specific action of the phytoconstituents against uropathogens are limited. *Vaccinium macrocarpon* Aiton (cranberry) is the best-studied home remedy for UTI. Some evidences suggest that proanthocyanins present in cranberry, prevent bacteria from adhering to the walls of the urinary tract, subsequently blocking the further steps of uropathogenesis. Probiotics such as *Lactobacillus* and *Bifidobacterium* are beneficial microorganisms that may act by the competitive exclusion principle to defend against infections in the urogenital tracts. Reports on potential vaccine agents and antibodies targeting the different toxins and effecter proteins are still obscure except uropathogenic *E. coli*.

**Conclusion:**

This review highlights some of the medicinal herbs used by aborigines to prevent or treat acute or chronic urinary tract infections, botanicals with established urobactericidal activity, clinical trials undertaken to compare the efficacy of cranberry products in UTI prevention, and other natural therapeutics reported for UTI.

## Background

Urinary tract infection (UTI) is a condition when any part of the urinary tract (urethra, bladder, ureter, and kidney) gets infected with bacteria or occasionally with fungus that evades the host defense barrier and colonize the urinary tract. The effect of UTI ranges from a mild self-limiting sickness to acute sepsis, with a mortality rate of 20-40% [1], which increases inexplicably with age. Both the sexes are prone to develop UTI with a female to male ratio of 2:1 in patients older than 70 years as compared to a 50:1 ratio in younger population [2]. It is the second most common infection after respiratory tract infections. Different methods are practiced to treat and prevent chronic and recurrent UTI, i.e., taking antibiotics, bioactive natural foods, using probiotics, and maintaining good personal hygiene, but still, they are yet to be addressed successfully. As UTI is generally caused by bacteria, they are most frequently treated with antibiotics. But, the type of medication and length of treatment depends on type of bacteria, its level of susceptibility, history, symptoms, and immune status of the patient.

It is not known, what percentage of people are now using alternative therapies, but certainly large numbers of women are drinking cranberry juice or using herbal remedies to enhance their immune status or taking probiotics to restore the normal vaginal flora, which usually gets disturbed after an antibiotic therapy. Vaccine development for organisms other than *E. coli* still remains obscure [3]. Cranberry, mannose, and probiotics are frequently used for recurrent UTI, and berberine and uva ursi are prescribed for acute UTI. Potassium salt supplements reduce dysuria by alkalinizing the urine. Application of estriol cream and supplement of vitamins A and C were considered to be effective to prevent UTI [4]. Generally, people drink plenty of water to flush out the infectious bacteria. Application of curd water around the urethra can help in getting rid of urinary burning sensation. This present review enlists some ethnobotanicals, which are reported to be beneficial for UTI and other urinary disorders. It covers a list of potential herbs with urobactericidal activity, the in vitro/in vivo and clinical trial studies reported to prove the efficacy of cranberry in treating UTI. It also represents the synopsis of relevant natural therapeutics, those are proven to be useful in both prevention and cure of urological disorders.

## Methods

Intense review of literature on the prevalence, mechanism of urinary tract infection, risk factors, preventive measures, and natural therapeutics for UTI were carried out using different databases like Google, Pubmed, and Sciencedirect. The keywords like the preventive and therapeutic role of different plants and their products in uropathogenesis, medicinal plants for acute and recurrent UTI, natural remedies, therapeutics for UTI, and anti-uropathogenic activity of medicinal plants, role of cranberry in acute and recurrent UTI were accessed from Medline, Google, Pubmed, and from different books, electronic, and printed journals, available in the library of Berhampur University, Utkal University, Institute of Life Sciences, and Regional Medical Research Center, Bhubaneswar, Odisha. The different keywords like urinary tract infection, uropathogenic bacteria, uropathogenesis, and UPEC are used in Google, Pubmed, and www.asm.org websites. The language chosen was English and both research and review articles were taken into account.

## Botanicals used for UTI

Therapeutic botanicals are defined as plants and their products with medicinal value. Indigenous plants are used for various ailments since time immemorial by mankind and probably we had learned this art from animals, since they have the inherent ability to use natural products for their different health ailments. These natural products are rich in diverse bioactive compounds, which form the basis for the development of new pharmaceuticals. There are immense advantages of using therapeutic botanicals like lesser side effects, more patient approval, less costly, and can be renewed naturally [5]. There are many reports that phytochemicals act as multi-drug resistance inhibitors/modulators that augment the effect of commonly used antibiotics [6, 7]. Diuretics like *Solidago* spp (goldenrod) herb, *Levisticum officinale* (lovage) root, *Petroselinum crispus* (parsley) fruit, and *Urtica dioica* (stinging nettle) increase urine volume in both healthy and people with urinary disorders that help in flushing out the probable threats. People, who consume antiseptic and anti-adhesive herbs like *Arctostaphylos uva-ursi* (uva ursi), *Juniperus* spp (Juniper) leaf, and fruit of *Vaccinium macrocarpon* (cranberry) excrete antimicrobial compounds, which may directly kill microbes or interfere with their adhesion to epithelial cells, thereby protecting against acute and chronic UTI [8]. The roots of *Mahonia aquifolium* (Pursh) Nutt. (Oregon grape) (Berberidaceae) and *Hydrastis canadensis* L. (Goldenseal) (Ranunculaceae) are rich in berberine. Berberine is an important drug against many bacteria and combat infections by preventing the bacteria (*E. coli* and *Proteus* species) from adhering to the host cell [9], which suggests their potent role in treating UTI.

Supplement of aqueous extract of corn (*Zea mays* L.) silk (outer thread-like part) to UTI patients significantly reduced the symptoms by reducing the number of RBCs, pus cells, and crystals in urine without any side effects [10]. It is rich in diverse therapeutic compounds [11]. Plants belonging to family Apiaceae, Fabaceae, Malvaceae followed by Asteraceae and Cucurbitaceae were found to be very effective against UTI [12]. Ethnomedicinal use of some plants against recurrent and chronic UTI is listed in Table 1.

**Table 1:** Directory of some important ethnomedicinal plants/plant parts used for UTI

Botanical name (family)	Parts used	Disorder/disease	Reference
*Adiantum lunulatum* Burm. f. (Pteridaceae)	Root	Blood discharge in urine	[13]
*Argemone mexicana* L. (Papaveraceae)	Root	Urinary trouble	[14]
*Clausena excavate* Burm. f. (Rutaceae)	Root	Urinary infection	[15]
*Cucumis melo* L. (Cucurbitaceae)	Epicarp	Kidney stone, urinary tract infection	[14]
*Cucumis sativus* L. (Cucurbitaceae)	Seed	Urinary tract infection	[16]
*Euphorbia thymifolia* L. (Euphorbiaceae)	Whole plant	Blood in urine	[17]
*Mimosa pudica* L.(Mimosaceae)	Root, leaf	Urinary infection, burning micturition	[18, 19]
*Asparagus racemosus* Willd. (Asparagaceae)	Roots	Urinary troubles	[20]
*Azadirachta indica* A. Juss. (Meliaceae)	Leaves	Urinary troubles	
*Cissampelos pareira* L. (Menispermaceae)	Roots, leaves	Urinary tract infection, diuretic	
*Crateva unilocularis* Buch.-Ham. (Capparaceae)	Leaves	Urinary diseases, kidney diseases	
*Malva verticillata* L. (Malvaceae)	Root	Urinary tract infection	
*Mangifera indica* L. (Anacardiaceae)	Branch	Urinary diseases, kidney diseases	
*Phyllanthus urinaria* L.(Euphorbiaceae)	Whole plant	Urinary problem	
*Tinospora sinensis* (Lour.) Merr. (Menispermaceae)	Whole plant	Urinary troubles, diuretic	
*Abutilon indicum* (L.) Sweet (Malvaceae)	Leaf	UTI, kidney stone	[21]
*Crateva nurvel* Buch-Ham. (Capparaceae)	Bark	UTI	
*Cyanodon dactylon* (L.) Persoon (Poaceae)	Root	Urolithiasis	
*Tribulus terrestris* L. (Zygophyllaceae)	Root, fruit	Kidney stone	
*Acacia farnesiana* (L.) Willd. (Fabaceae)	Roots	Burning sensation in the urinary tract, UTI oliguria and polyuria	[22]
*Acanthus ilicifolius* L. (Acanthaceae)	Roots	Unclear urine in women	
*Acrostichum aureum* L. (Pteridaceae)	Leaves	Unclear urine in women, UTI	
*Ageratum conyzoides* L. (Asteraceae)	Leaves, roots	UTI	
*Caesalpinia nuga* (L.) Aiton (Caesalpiniaceae)	Plant juice, roots, fruit	Urinary tract disorder, oliguria, and polyuria	
*Clitoria ternatea* L. (Fabaceae)	Leaves	Urinary tract problems	
*Elephantopus scaber* L. (Asteraceae)	Roots	Difficulties in urination	
*Hemidesmus indicus* (L.) R. Br. (Asclepiadaceae)	Leaves	Urinary tract infections	
*Mimosa pudica* L. (Mimosaceae)	Roots, barks	Urinary problems	
*Moghania macrophylla* (Willd.) Kuntze (Fabaceae)	Root	Retrograde ejaculation, painful urination	
*Melastoma malabathricum* L. (Melastomataceae)	Roots, leaves	Burning sensations in the urinary tract, painful urination, oliguria, and polyuria	
*Nymphaea nouchali* Burm. f. (Nymphaeaceae)	Root tops	Urinary ailments	
*Oroxylum indicum* (L.) Kurz (Bignoniaceae)	Bark, fruit	Difficulties in urination, burning sensation, red urination, polyuria, lower abdominal pain	
*Stephania japonica* (Thunb.) Miers (Menispermaceae)	Vines	UTI, diuretic	
*Urena lobata* L. (Malvaceae)	Roots, leaves, bark, flowers	Urinary trouble, burning sensations in the urinary tract	
*Zizyphus oenoplia* (L.) Mill. (Rhamnaceae)	Root	Urinary disorders	
*Santalum album* L. (Santalaceae)	Tender twig	UTI	[23]

## Botanicals with anti-uropathogenic activity

Few Jordanian plants were reported to have antibiotic resistance-modifying activity against MDR *E. coli*. Especially, methanol extracts of the plant parts improved the effects of cephalexin, doxycycline, neomycin, chloramphenicol, and nalidixic acid against both the standard and resistant strains of *E. coli*. Extracts of *Anagyris foetida* L. (Fabaceae) and *Lepidium sativum* L. (Apiaceae) had differential activity against the standard and resistant strains as it decreased the activity of amoxicillin against the standard strain but increased the activity against resistant strains. Edible plants like *Gundelia tournefortii* L. (Asteraceae), *Eruca sativa* Mill. (Brassicaceae), and *Origanum syriacum* L. (Lamiaceae), augmented clarithromycin activity against the resistant *E. coli* strain. Perhaps these antibiotics and plant extracts may be prescribed together to treat infections caused by MDR *E. coli* [24]. There are numerous reports for the anti-uropathogenic and urobactericidal activities of various plants and their products, which are listed in Table 2.

**Table 2:** List of medicinal plants with anti-uropathogenic potential

Plant name (family)	Extract/part used	Name of microorganism	Reference
*Ocimum gratissimum* L., *Salvia officinalis* L. (Lamiaceae); *Cymbopogon citratus* (DC.) Stapf (Poaceae)	Essential oil	*Klebsiella pneumoniae*; *K. oxytoca*; *E. coli*; *Enterobacter aerogenes*; *Morganella morganii*; *P. mirabilis*	[25]
*Mangifera indica* L. (Anacardiaceae)	Water and ethanol extract of seed kernel	*Staphylococcus aureus*	[26]
*Zinziber officinale* Roscoe (Zinziberaceae); *Punica granatum* L. (Lythraceae)	Ethanol extract of rhizome and seed, respectively	*E. coli*	[27]
*Ocimum gratissimum* L. (Lamiaceae)	Ethanol extract of leaf	*E. coli*; *P. mirabilis*; *S. aureus*; *Pseudomonas aeruginosa*; *Candida albicans*	[28]
*Carica papaya* L. (Caricaceae)	Water, chloroform, ethanol extract of leaves	*K. pneumoniae*; *E. coli*; *P. mirabilis*	[29]
*Ibicella lutea* (Lindl.) Van Eselt. (Martyniaceae)	Plant extract	*P. mirabilis*	[30]
*Allium sativum* L. (Liliaceae)	Allicin from clove and leaf	*E. coli*; *S. aureus*	[31]
*Rhizophora apiculata* Blume; *R. Mucronata* Lam.; *Bruguiera cylindrical* (L.) Blume; *Ceriops decandre* (Griff.) W.Theob. (Rhizophoraceae); *Avicennia marina* (Forssk.) Vierh. (Acanthaceae)	Ethanol extract of hypocotyl, bark, collar, and flower	*E. coli*; *K. pneumonia*; *P. aeruginosa*; *S. aureus*; *Enterobacter* sp*.*	[32]
*Coccinia grandis* (L.) Voigt (Cucurbitaceae)	Water, acetone, ethanol extract of leaves	Uropathogenic *E. coli* (UPEC)	[33]
*Coleus aromaticus* Lour.; *Ocimum sanctum* L. (Lamiaceae)	Essential oil	*E. coli*; *S. aureus*; *K. pneumonia*; *Klebsiella oxytoca*; *Proteus vulgaris*; *P. mirabilis*; *P. aeruginosa*	[34]
*Clitoria ternatea* L. (Fabaceae); *Achyranthes aspera*. L. (Amaranthaceae)	Leaf extract	*E. coli*; methicillin resistant *S. aureus*; *S. aureus*; *P. aeruginosa*; *K. pneumonia*; *Citrobacter diverses*; *Serratia liquefaciens*; *C. albicans*	[35]
*Moringa oleifera* Lam. (Moringaceae)	Leaf extract	*P. mirabilis*	[36]
*Azadirachta indica* L. (Meliaceae); *Tinospora cordifolia* (Willd.) Miers (Menispermaceae); *Euphorbia hirta* L. (Euphorbiaceae); *Cassia javanica* L. (Fabaceae); *Phyllanthus niruri* L. (Euphorbiaceae); *Asparagus racemosus* Willd. (Asparagaceae); *Eupatorium triplinrrve* Blume (Asteraceae)	Chloroform, methanol, acetone, ethanol extract	*P. aeruginosa*; *Staphylococcus epidermis*; *Serratia marcescens*; *Enterobacter*; *Citrobacter*	[37]
*Piptochaetium montevidense* (Spreng.) Parodi (Poaceae); *Bulbostylis cappilaris* (L.) Kunth ex C.B. Clarke (Cyperaceae); *Juncus capillaceus* Lam. (Juncaceae)	Plant extract	*E. coli*; *K. pneumoniae*	[38]
*Cymbopogon citrates* (DC.) Stapf (Poaceae); *Syzygium aromaticum* (L.) Merr. & L.M. Perry (Myrtaceae)	Essential oil	*C. albicans*	[39]
Seagrass (*Halodule pinifolia*) (Miki) Hartog; *Cymodocea rotundata* Asch. & Schweinf. (Cymodoceaceae)	Aqueous methanol (1:4) extract of fresh leaves	*E. coli*; *S. saprophyticus*; *P. aeruginosa*; *K. pneumonia*; *P. mirabilis*; *Serratia* sp	[40]
*Betula pendula* Roth. (Betulaceae); *Equisetum arvense* L. (Equisetaceae); *Herniaria glabra* L. (Caryophyllaceae); *Galium odoratum* (L.) Scop. (Rubiaceae); *Urtica dioica* L. (Urticaceae); *Vaccinium vitis-idae* L. (Ericaceae)	Aqueous extract	*E. coli*	[41]
*Camellia sinensis* (L.) Kuntze (Theaceae)	Leaf extract	*E. coli*	[42]
*Aerva lanata* (L.) Juss. ex Schult. (Amaranthaceae); *Biophytum sensitivum* (L.) DC. (Oxalidaceae); *Boerhavia diffusa* L. (Nyctaginaceae); *Myristica fragrans* Houtt. (Myristicaceae)	Petroleum ether, chloroform, methanol, water extract of whole plant, and nutmeg nuts	*E. coli*; *S. aureus*; *S. viridians*; *P. aeruginosa*; *K. pneumoniae*	[43]
*Punica granatum* L. (Lythraceae); *Stevia rebaudiana* (Beroni) Bertoni; *Allium sativum* L. Amaryllidaceae	Alcohol or water extract; basil oil, geranium oil, lemon grass oil, Japanese mint oil	*P. mirabilis*; *P aeruginosa*; *Acinetobacter*; *Serratia*; *Klebsiella*	[44]
*Mangifera indica* L. (Anacardiaceae)	Methanol extract of flower	UPEC	[45]
*Pimenta dioica* (L.) Merr. (Myrtaceae); *Anacardium occidentale* L. (Anacardiaceae)	Leaf and bark extract	*E. coli*; *E. faecalis*; *P. aeruginosa*; *S.aureus*; *K. pneumoniae*	[46]
*Salvia santolinifolia* Boiss. (Lamiaceae)	Essential oil	*K. pneumoniae*; *P. mirabilis*; *P. vulgaris*	[47]
20 plants (*Betula*; *Urtica*; *Orthosiphon*; *Zea mays*; *Agropyron repens*, etc.)	Leaves	UPEC	[48]
*Tribulus terrestris* L. (Zygoplyllaceae); *Cinnamom verum* J. Presl. (Lauraceae); *Punica granatum* L. (Lythraceae)	Aqueous and ethanol extract of dried plant	*E. coli*; *K. pneumoniae*; *S. aureus from pregnant women*	[49]
*Camellia sinensis* (L.) Kuntze (Theaceae)	Leaf extract	*E. coli*	[50]
*Callistemon lanceolatus* DC. (Myrtaceae)	Petroleum ether, chloroform, ethanol, methanol seed extract	*S. aureus*; *A. baumani*; *C. feundii*; *E. faecalis*; *E. coli*; *K. pneumoniae*	[51]
*Anacardium occidentale* L. (Anacardiaceae)	Fruit juice	*P. aeruginosa*; *E. faecalis*; *E. coli*	[52]
*Hibiscus sabdariffa* L. (Malvaceae)	Calyx extract	*C. albicans*	[53]
*Senna sophera* (L.) Roxb synonym *Cassia sophera* L. (Fabaceae)	Alcoholic leaf extract	*E. coli*, *K. pneumoniae*, *P. mirabilis*, *P. aeruginosa*, *Citrobacter freundii*, *Enterococcus faecalis*, and *S. saprophyticus*	[54]
*Ocimum suave* Willd. (Lamiaceae)	Essential oil	*S. aureus*; *K. pneumoniae*; *E. faecalis*; *P. aeruginosa*; *Morganella morgani*; *Enterobacter*; *Acinetobacter*; *Citrobacter*	[55]
*Acanthus montanus* (Nees) T. Anderson (Acanthaceae); *Aspilia africana* C.D. Adams (Asteraceae); *Desmodium velutinum* (Willd.) DC (Fabaceae)	Ethanol and aqueous extract of leaves	*E. coli*, *P. aeruginosa*, *S. aureus*	[56]
*Allium sativum* L. (Liliaceae); *Cinnamomum verum* J. Presl (Lauraceae); *Syzygium aromaticum* (L.) Merrill & Perry (Myrtaceae); *Terminalia arjuna* (Roxb.) Wight & Arn. (Combretaceae); *Zingiber officinale* Roscoe (Zingiberaceae)	Ethanol and aqueous extract of rhizome, bark, flower, bark, rhizome, respectively	*E. coli*, *P. aeruginosa*, *P. vulgaris S. aureus*, *K. pneumoniae*	[57]
*Syzygium aromaticum* (L.) Merr. & L. M. Perry (Myrtaceae), *Glycerrhiza glabra* L. (Fabaceae), *Laurus nobilis* L. (Lauraceae), and *Brassica rapa* L. (Brassicaceae)	Methanol extract of buds, roots, leaves, seeds, respectively	*E. coli*, *Acinetobacter baumannii*, and *P. aeruginosa*	[58]
*Hemidesmus indicus* R. Br. (Asclepiadaceae)	Methanol extract of root	*E. coli*, *K. pneumoniae*	[59]

## Cranberry: a potent uroprotective agent

For centuries, cranberries have been used as a treatment for urinary tract diseases and its antibacterial activity was reported long back [60]. It contains > 80% water, 10% carbohydrates (glucose and fructose) [61], and other phytoconstituents like anthocyanins, flavonoids, terpenoids, catechins, organic acids (citric acid, malic acid, and quinic acid, etc.) with small amount of ascorbic acid, benzoic acid, glucuronic acids [62]. Quinic acid was suggested to be responsible for excretion of hippuric acid in urine in large amounts, which is an antibacterial agent and also has the ability to acidify the urine [63, 64]. Moreover, the elucidation of the UTI pathogenesis has opened a new vista to understand the mode of action of cranberry as an anti-adhesive prophylactic and therapeutic agent for UTI [65].

*Escherichia coli* strains isolated from urine (UPEC) attached three times more efficiently to uroepithelial cells than *E. coli* isolated from other experimental sources like stool, sputum, or wound. This proves a unique population of *E. coli* strain responsible for UTI [66]. Antiadherence activity against gram-negative bacteria isolated from urine and other medical sources was observed in volunteers administered with cranberry juice cocktail or urine and uroepithelial cells obtained after drinking the cocktail, which proves its efficacy in treating UTI [66]. Consumption of different cranberry products helped young and elderly women in preventing and protecting them against UTI [67].

**Fig. 1:**
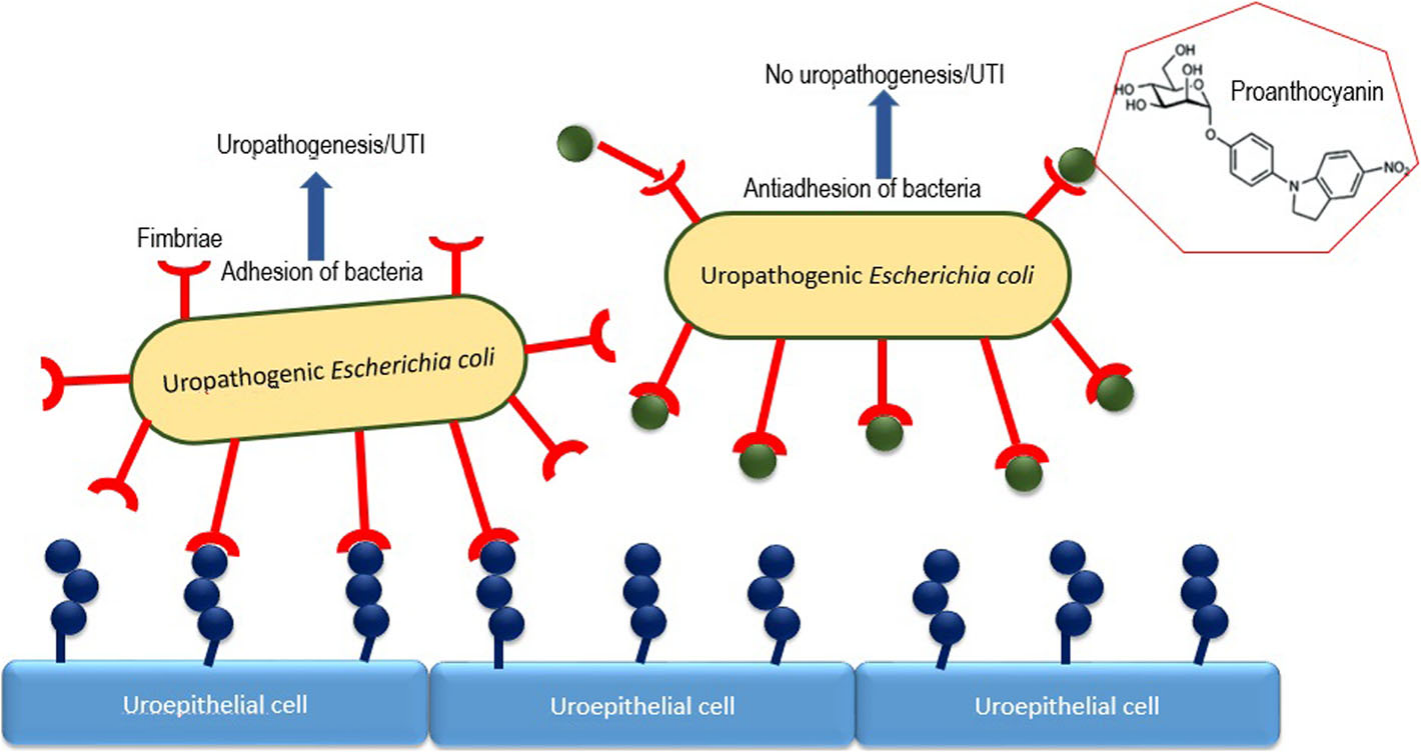
Type 1 or P-fimbriae inhibitors (e.g., proanthocyanins) are shown as green balls interfering with binding of bacterial fimbriae to uroepithelial cell

The anthocyanidin/proanthocyanidin biocompounds present in cranberry are reported often to be potent antiadhesive compounds. Since cranberry inhibits the adhesion of type I and P-fimbriated uropathogens (e.g., uropathogenic *E. coli*) to the uroepithelium, thus, weaken colonization and succeeding infection [68]. Figure 1 depicts the molecular mechanism of antiadhesive property of proanthocyanidins. Due to lack of proper standardization of cranberry products, it becomes extremely complicated to compare products or correlate the results [69]. The in vitro and in vivo studies were summarized in Table 3.

**Table 3:** In vitro/in vivo activity of cranberries against UTI causing bacteria

Study design	Dose	Microorganism	Result	Reference
In vitro antiadhesion activity of cranberry (PAC)	10-50 μg/ml	UPEC	PAC derived from cranberry and blueberry was effective.	[70]
In vitro antiadhesion activity of cranberry (PAC)	60 μg/ml	UPEC	A-linked PAC were more effective than B-linked.	[71]
Antiadhesion activity of cranberry vs raisins	42.5 g	UPEC	25-50% of reduction in adherence in cranberry gr. None in control or raisin gr.	[72]
In vitro antiadhesion activity of cranberry juice	27% cranberry juice (250 or 750 ml)	*E. coli*	45% and 62% decrease in bacterial adhesion to human epithelial cell line in bacteria growing in urine of volunteers administered with 250 and 750 ml of cranberry juice, respectively.	[73]
Anti-adhesion activity and prevention of oxidative stress of dried cranberry juice in young women	Dried cranberry juice (400 mg or 1200 mg per day) for 56 days	UPEC	Inhibition of adherence in UPEC with no urine acidity observed in volunteers consuming 1200 mg/day. No effect observed at 400 mg/day.	[74]
Anti-adhesion activity of cranberry PAC against bladder and vaginal epithelial cells	5 to 75 μg/ml of PAC isolated from cranberry powder or extract	*E. coli*	50 μg/ml of PAC reduced the mean adherence of *E. coli* IA2 to vaginal epithelial cells from 18.6 to 1.8 and bladder epithelial cell from 6.9 to 1.6 bacteria per cell	[75]
In vitro and in vivo antibacterial and anti-adhesion activity of urine, after cranberry consumption in volunteers	36 (1 capsule) or 108 mg (3 capsules) of cranberry or placebo per day	*E. coli*	Better anti-adherence to bladder cell and virulence reduction in *E. coli* infecting worms when bacteria cultured in urine of volunteer administered with three capsules (108 mg/day) then single capsule (36 mg/day).	[76]
Anti-adhesion activity of cranberry juice	Juice or PAC of 0, 64, 128 and 345.8 mg/ml	*E. coli*	*E. coli* grown in the presence of PAC repressed adhesion from 50.2 to 7.9 bacteria/cell by altering its surface properties and the effect was reversible.	[77]
Antimicrobial activity of urine after cranberry consumption in volunteers	275 mg of dry, whole cranberries or 25 mg of concentrated, dry cranberries	*E. coli, K. pneumonia* and *C. albicans*	≥ 50% reduction in bacterial number when grown in urine of volunteers after cranberry consumption was found to be 35% (*E. coli*), 65% (*K. pneumoniae*), and 45% (*C. albicans*).	[78]
Bacterial anti-adhesion activity of urine collected from cranberry powder administered volunteers	Cranberry capsule of 0, 18, 36, or 72 mg of PAC equivalents per day	*E. coli*	Dose-dependent decrease in adhesion to bladder cell and reduction in virulence of UPEC in *C. elegans* model	[79]
In vitro anti-adhesion assay in T24 cell line and in vivo virulence assay in *C. elegans* model	PAC (6-120 mg) plus propolis (170-340 mg) powder	*E. coli*	Synergistic activity of propolis and proanthocyanidins	[80]
In vitro activity of PAC	4–1024 mg/L	*C. albicans*	Reduction in biofilm formation due to anti-adherence properties and/or iron chelation at a dose of ≥ 16 mg/L PAC	[81]
In vitro activity of A2-linked PAC	15-100 μg/mL	UPEC, *P. mirabilis*	Up to 75% reduction of UPEC and *P. mirabilis* adhesion to HT1376 cell line vs. control. Also drop in motility and urease activity in *P. mirabilis*.	[82]
*In vitro and in vivo activity of PAC*	100 μg/mL	*P. aeruginosa*	Cranberry PACs significantly disrupted the biofilm formation	[83]
In vitro activity of oligosaccharides	0.625-10 mg/mL	*E. coli*	Reduced biofilm formation by over 50% in pathogenic form and over 60% in nonpathogenic *E. coli*	[84]
Antiadhesive activity of phenolic compounds and their metabolites derived from cranberry	100–500 μM	UPEC	All the metabolites showed anti-adhesive activity but procyanidin A2, significantly reduced UPEC adherence to uroepithelium at 500 μM (51.3%).	[85]
Ex vivo and in vitro antiadhesive activity of PAC and PAC free extract	Standard cranberry extract with 1.24% PAC for ex vivo and 21% PAC for in vitro study	UPEC	40-50% suppression of UPEC adhesion to human T24 bladder cells. PAC free extract did not influence biofilm and curli formation in UPEC.	[86]
In vivo a*ctivity of cranberry juice and its organic acids in mice*	*Cranberry juice/bioactive compounds taken for 7 days*	UPEC	Reduction of bacterial number in the bladder of mice drinking fresh cranberry juice, organic acids or both.	[87]

The recurrence of UTI rates was reduced up to 35% in young to middle-aged women, after the use of cranberry-based compounds. But, in groups with complicated UTI (i.e., young and elderly patients, or patients with neurogenic bladder or with chronic indwelling catheters), the potency of cranberry was unclear. However, these compounds cannot be taken for a longer duration as they have some undesirable effects like weight gain, gastrointestinal problems, and harmful interactions with other drugs [69]. Clinical trials were often complicated and results are not satisfactory in patients with complicated UTI, whereas, cranberry uptake significantly prevented acute cystitis in high-risk females [88]. The clinical trials undertaken with cranberry were summarized in Table 4.

**Table 4:** Clinical trials of cranberry products for UTI prevention in different populations

Experimental design	Dose	*N*	Result	Reference
Randomized, double-blind, placebo-controlled trial	Cranberry juice of 300 ml/day or placebo	153 elderly women	UTI incidence 15% in cranberry group and 28.1% in placebo group (difference is non-significant)	[89]
Randomized, single-blind cross over study	15 ml juice/kg or water placebo	21 patients with neuropathic bladder	9 patients taking cranberry juice and 9 patients taking water showed lowered infection, rest 3 were indifferent.	[90]
Randomized, double-blind, crossover trial	Cranberry capsules of 400 mg	19 female having recurrent UTIs	UTI incidences were 2.4/subject/year in cranberry group and 6.0/subject/year in placebo, 47.4% of withdrawal rate.	[91]
Double-blind placebo controlled with crossover	60 ml/day of cranberry juice or placebo	15 children under intermittent catheterization	Differences between groups are nonsignificant for bacteriuria or UTI.	[92]
Randomized, double-blind, placebo-controlled	50 ml of cranberry-lingonberry juice (7.5 g), *Lactobacillus* GG 100 ml/day or placebo	150 young women with previous UTI	Recurrence rate of UTI reduced in cranberry group, 20% less UTI in cranberry group.	[93]
Randomized, double-blind, placebo-controlled	Cranberry juice 250 ml or its tablets	150 women with recurrent UTIs	Incidence of UTI—30% in juice, 39% in tablets group and 72% in placebo	[94]
Randomized, double-blind, placebo-controlled	Cranberry capsules of 8 g or placebo	135 patients with complicated UTI (multiple sclerosis generated neurogenic bladder)	34.6% UTI in cranberry group and 32.4% on placebo, no significant difference between the groups and also under intermittent catheterization.	[95]
Randomized, double-blind, placebo-controlled	Cranberry capsules of 1 g or placebo	74 patients with neurogenic bladder induced by spinal cord injury	Insignificant differences in bacteriuria, pyuria, or symptomatic UTIs between the groups, 35% withdrawal rate	[96]
Double-blind, placebo controlled with crossover	400 mg of cranberry tablets for 4 weeks or placebo	37 patients with neurogenic bladder due to spinal cord injury	43% of withdrawal rate and no difference were observed between the cranberry and the placebo group.	[97]
Randomized, double-blind, placebo-controlled	25% of cranberry juice (150 ml) and placebo	376 in door old patients (> 60 years)	3.7% of UTI incidences in cranberry group of 7.4% with placebo 31% withdrawal rate	[98]
Double-blind, randomized, placebo-controlled	1st group—methenamine hippurate (MH), 2nd—cranberry (800 mg), 3rd—cranberry + MH, and 4th—placebo	305 patients with spinal cord injury resulted neurogenic bladder	No differences for symptomatic UTI groups to placebo	[99]
Randomized, double-blind, placebo-controlled trial	Group A—240 mg of 27% cranberry juice 3 times/day or group B—240 mg daily once or group C—placebo	188 pregnant women of 16 weeks gestation	No significant differences in UTI occurrence between the groups. Withdrawal rate of 38.8% (A, 50.7%, B, 39.7%, C, 55.5%)	[100]
Randomized, double-blind, placebo-controlled trial	Cranberry extract tablet for 6 months	47 spinal cord injured patients	0.3 UTI per year in cranberry group vs 1.0 UTI per year in placebo.	[101]
Randomized, double-blind, placebo-controlled trial	cranberry extract (500 mg) or trimethoprim (100 mg)	137 women with recurrent UTIs—age 45 years	25 UTIs in cranberry group and14 in trimethoprim group	[102]
Randomized controlled trial	Cranberry-lingonberry juice 50 ml/day, *Lactobacillus* GG 100 ml, 5 days/month or placebo	84 girls with recurrent UTIs	UTIs incidence 18.5% in 1st group, 42.3% in 2nd, and 48.1% in placebo	[103]
Randomized, double-blind, placebo-controlled trial	27% cranberry juice (8 oz.)	319 young women with UTI history	UTI recurrence rates—19.3% for cranberry group and 14.6% for placebo	[104]
Randomized, double-blind, placebo-controlled trial	Cranberry juice	263 children cranberry (*n* = 129), placebo (*n* = 134)	0.1% UTI episodes lower in cranberry gr.	[105]
Randomized, double-blind, placebo-controlled trial	200 mg of cranberry	370 prostate cancer patients	8.7% UTI in cranberry group, 24.2% in placebo (36% reduction in UTI)	[106]
Randomized, double-blind, placebo-controlled trial	Cranberry juice 4, 8 oz/daily, or placebo	176 patients (120 to cranberry juice and 56 to placebo)	0.29 UTI in cranberry juice group and 0.37 in the placebo group. P-fimbriated UPEC isolation was 43.5% (10 of 23) in cranberry juice group, 80.0% (8 of 10) in placebo group during the study period	[107]
Randomized, double-blind, placebo-controlled trial	3 capsules of PAC daily for 30 days (108 mg, 72 mg, 36 mg)	80 women	Dose-dependent reduction in bacteriuria and pyuria	[108]
Modified observational study	Sweetened dried cranberry (SDC) of one serving daily for 14 days	20 women with recurrent UTIs	Mean UTI rate per six months decreased significantly, no UTI observed in > 50% of the patients up to 6 months of SDC consumption	[109]
Randomized, double-blind, placebo-controlled multicenter trial	Capsules of cranberry and placebo were taken twice daily for 1 year	928 women of high and low risk group	Incidence of UTI reduced in cranberry than placebo group (62.8 vs 84.8 per 100 person-years in UTI high risk group). No difference observed in low UTI risk group	[110]
Randomized, double-blind, placebo-controlled trial	Two cranberry juice capsules twice daily for 6 weeks or placebo	160 women undergoing gynecological surgery involving urinary catheterization (80 + 80)	19% UTI incidence in cranberry group compared to 38% in placebo group	[111, 112]
Randomized, double-blind, placebo-controlled trial	500 mg of whole cranberry fruit powder for 6 months or placebo	Cranberry (*n* = 89) or a placebo group (*n* = 93)	UTI occurrence significantly lowered 10.8% vs 25.8% in cranberry and placebo group, respectively	[113]
Randomized, double-blind, placebo-controlled trial	240 ml of cranberry juice per day for 24 weeks or placebo	Cranberry (*n* = 185) or a placebo (*n* = 188)	UTI occurrence significantly lowered 21% vs 36% in cranberry and placebo group, respectively	[114]

## *Cinnamom verum* J. Presl. (cinnamon): a potent botanical for complicated UTI

Chronic recurrent UTI was resulted in patients with urinary catheters due to biofilm formation by MDR UPEC. Trans-cinnamaldehyde (0%, 1%, 1.25%, or 1.5%) was reported to prevent UPEC biofilm formation both on plate culture and indwelling catheters. When trans-cinnamaldehyde was used in catheter lock solution, it inactivated UPEC biofilm formation on catheters. Since the test concentrations had no cytotoxic effects on human bladder epithelial cells, it can be used as a surface coating for catheters or in catheter lock solution to prevent UTI [115]. Trans-cinnamaldehyde significantly reduced uroepithelial cell attachment and invasion by UPEC by inhibiting the expression of major genes associated with its attachment and invasion to host tissue [116]. These findings support the use of cinnamon as a natural remedy for UTI.

## *Arctostaphylos uva-ursi* (L.) Spreng (bearberry)

*Arctostaphylos uva-ursi* (uva ursi), also known as bearberry or upland cranberry, is a useful herb for bladder infection. Bearberry leaves and preparations made from them have significant antibacterial activity (especially against *E. coli*) and astringent activity due to its arbutin content and diuretic properties. In a double-blind study of 57 women, five of twenty-seven women had a recurrence in the placebo group while none of thirty women had a recurrence in the uva ursi group after 1 year [117]. Schindler et al. reported that the total amount of urinary excretion of arbutin metabolites (hydroquinone) remained same in all the three groups, after the administration of a single oral dose of bearberry leaves extract or film-coated tablets or an aqueous solution in a randomized crossover study (*n* = 16) [118].

## Probiotics

Probiotics are helpful in establishing and maintaining normal ecology of the vagina, urethra, and bladder and a proper bladder pH and preventing recurrent UTI, which was supported by various in vivo and in vitro studies*.* Lactobacilli are present predominantly in the urogenital flora of healthy reproductive-aged women. But, the flora is disturbed following long term antibiotic administration and post menstruation temporarily and in post-menopausal women permanently. Supplement of *Lactobacillus rhamnosus GR-1 and Lactobacillus fermentum RC-14* appears to be most effective in reducing the risk of intestinal and urogenital infections [119]. The antagonistic activity of five probiotic lactobacilli (*L. rhamnosus*, *L. fermentum*, *L. acidophilus*, *L. plantarum,* and *L. paracasei*) and two bifidobacteria (*Bifidobacterium lactis*, *B. longum*) against six target pathogens were estimated using different assays. Pyelonephritic *E. coli* was highly suppressed by *L. rhamnosus* and both bifidobacterial strains [120]. One hundred thirty-nine women (mean age: 30.5 years) with acute UTI were compared with 185 women of similar age with no episodes of UTIs for 5 years. Frequent consumption of fresh juices, especially berry juices, and fermented milk products containing probiotic bacteria decreased the risk of recurrence of UTI in fertile women. So, dietary supplements can be used to prevent UTI [121].

Preincubation of the uroepithelial cells with *Lactobacillus* bacterial cell wall fragments inhibited the adherence and colonization of gram-negative uropathogens either completely or partially, which prevented the onset of UTI in female rats. Since the lipoteichoic acid present in the bacterial cell wall is responsible for the adherence of the *Lactobacillus* cells to uroepithelial cells but its steric hindrance blocked the adherence of uropathogens [122, 123]. Seven strains of lactic acid bacteria (*L. paracasei*, *L. salivarius*, two *Pediococcus pentosaceus* strains, two *L. plantarum* strains, and *L. crispatus*) and their fermented probiotic products exhibited clear zones of inhibition against UPEC. This suggests their potential role in adjuvant therapy for prevention and treatment of UTI. The growth of UPEC strains was significantly inhibited after co-culture with lactic acid bacteria and probiotic products in human urine. Oral administration of probiotic products also abrogated the number of viable UPEC in the urine of UPEC-challenged BALB/c mice [124].

## Vaccines

Adhesin-based vaccines were very effective in blocking host–pathogen interactions, thereby preventing the establishment of disease [125–127]. In addition to the UPEC adhesins (i.e., pili, fimbriae), adhesins from *P. mirabilis*, and *E. faecalis* were also reported as vaccine targets [128]. Vaccination with HlyA (UPEC pore-forming toxin) reduced the rate of renal scaring compared to controls, though it could not prevent UPEC colonization of the kidneys [129]. Several urease inhibitors, i.e., acetohydroxamic acid (AHA), phosphoramidites, benzimidazoles have been used as potent drugs for UTI treatment against urease producing bacterial species like *P. mirabilis* and *S. saprophyticus* [130]. Pilicides (type 1 pilus assembly inhibitor) and mannosides (pili function inhibitor) block UPEC colonization, invasion, and biofilm formation and prevent UTI [131, 132].

## Discussions

Antibiotics are frequently used to treat and prevent acute and recurrent UTI, but their repeated use can result in dysbiosis of vaginal and intestinal normal flora, as well as antibiotic resistance due to the high mutation ability and horizontal gene transfer capability of different pathogens. Moreover, different mechanisms are used by uropathogens for survival in the bladder under stresses such as starvation and immune responses. Uropathogens undergo morphological changes, invade uroepithelial cells, and form biofilms to persist and cause recurrent infections. Extracellular DNA, exopolysaccharides, pili, flagella, and other adhesive fibers create a niche for a bacterial community that is secluded from antimicrobial agents, immune responses, and other stresses [133]. Thus, it is high time to seek alternative methods for the prevention and treatment of UTIs.

Diuretic botanicals like *Asparagus officinalis* L. (asparagus), *Betula spp.* (birch) *Elymus repens* (L.) Gould (synonym: *Agropyron repens*) (couch grass), *Solidago virgaurea* L. (goldenrod), and *Equisetum arvense* L. (horsetail) work against UTI by increasing urinary volume and supposedly flushing bacteria out of the urinary tract. Ayurvedic herbs like *Tribulus terrestris* L., *Boerhavia diffusa* L., *Tinospora cordifolia* (Willd.) Miers, and *Santalum album* L. are used since time immemorial for UTI in India. The tribes of Odisha state, India, use the roots of *Adiantum lunulatum* Burm. f, *Argemone mexicana* L., *Clausena excavata* Burm. f, *Mimosa pudica* L., epicarp of *Cucumis melo* L., and seeds of *Cucumis sativus* L. for UTIs. These herbs have proven anti-uropathogenic activities, which were reported enormously by different researchers. However, reports on anti-uropathogenic activity of specific phytoconstituents or their mode of action at the molecular level on uropathogens like enzyme or protein inhibition or degradation, cell membrane, or cell wall disruption or dysfunction of other vital organs of uropathogens are limited. Though the herbal remedies are considered safe to use without any significant side effects yet they are slow in action to be effective in serious acute infections, but they are more effective in preventing recurrence and safeguarding against the post-infectious sequelae.

The safety and efficacy of a product containing two probiotic strains of Lactobacilli plus cranberry extract was reported for impeding recurrent UTIs in pre-menopausal adult women. After 26 weeks, in a randomized, double-blind, placebo-controlled pilot study, a significantly lower number of women experienced recurrent UTIs (9.1 vs 33.3%), those who were administered with the product as compared to placebo [134]. In another study, the efficacy and safety of standardized cranberry capsules as prophylaxis in children with recurrent UTI was reported, where children on cranberry compared to the control group experienced significantly lower percentage of recurrent UTIs, with no side effects. A declined trend of *E. coli* infections was observed in the cranberry group (83.3% vs. 66.6%), though it was not significant (*p* = 0.28) [135].

Root extract of *Hemidesmus indicus* R. Br. (Indian sarsaparilla) (Asclepiadaceae) and seed extract of *P. granatum* (pomegranate) were reported to have urobactericidal activity against different uropathogens, clinically isolated from patients suffering from urinary tract infections, i.e., *Escherichia coli*, *Enterococcus faecalis*, *Staphylococcus aureus*, and *Klebsiella pneumonia* [59, 136, 137]. Along with the presence of therapeutic antioxidants, i.e., phenolic compounds, tannins, steroids, terpenes, coumarins, and flavonoids, the extracts were found to be rich in natural glycosides, which are supposed to act as molecular decoys to prevent adhesion of pathogenic bacteria to host cell, thereby inhibiting the future pathogenesis. However, further research is required to confirm it. Till date, there are many reports on scientific evaluations and clinical trials of natural therapeutics for UTI, but they have serious limitations in study design and data interpretation. Most of the products mentioned in this review are based on “in vitro” studies; therefore, more clinical trials should be undertaken in order to assess the efficacy of these alternative preventions and therapeutic methods in humans.

## Conclusion

Uroprotective role of cranberry was reported by maximum researchers, yet they suffer from serious drawbacks and fail to prove that cranberry use can prevent or treat acute and recurrent UTI. So, further investigation should focus on the molecular action of various phytochemicals present in cranberry and other potential berries against different uropathogens and uropathogenesis. Supplementation of probiotics was also proven to be effective in both acute and recurrent UTI. However, scientific validation with efficient clinical trial reports will strengthen the practice of using these traditional resources, which will help us in preventing these common yet very discomforting ailments.

## Data Availability

All data generated or analyzed during this study are included in this published article.

## Abbreviations

MIC: Minimum inhibitory concentration
MDR: Multidrug resistant
PAC: Proanthocyanidine
UTI: Urinary tract infection
UPEC: Uropathogenic *Escherichia coli*
